# Radiation Synthesis of High-Temperature Wide-Bandgap Ceramics

**DOI:** 10.3390/mi14122193

**Published:** 2023-11-30

**Authors:** Victor Lisitsyn, Aida Tulegenova, Mikhail Golkovski, Elena Polisadova, Liudmila Lisitsyna, Dossymkhan Mussakhanov, Gulnur Alpyssova

**Affiliations:** 1 Department of Materials Science, Engineering School, National Research Tomsk Polytechnic University, 30, Lenin Ave., Tomsk 634050, Russia; elp@tpu.ru; 2 Institute of Applied Science & Information Technology, Almaty 050042, Kazakhstan; 3 National Nanotechnology Laboratory of Open Type (NNLOT), Al-Farabi Kazakh National University, 71, Al-Farabi Ave., Almaty 050040, Kazakhstan; 4 Budker Institute of Nuclear Physics, SB RAS, 11, Lavrentiev Ave., Novosibirsk 630090, Russia; golkovski@mail.ru; 5 Department of Physics, Chemistry and Theoretical Mechanics, Tomsk State University of Architecture and Building, 2, Solyanaya Sq., Tomsk 634003, Russia; lisitsyna@mail.ru; 6 Department of Technical Physics, L.N. Gumilyov Eurasian National University, Astana 010000, Kazakhstan; dos_f@mail.ru; 7 Department of Radiophysics and Electronics, Karaganda Buketov University, Karaganda 100028, Kazakhstan; gulnur-0909@bk.ru

**Keywords:** radiation synthesis, refractory dielectric materials, luminescence, high-power electron flux, ceramics

## Abstract

This paper presents the results of ceramic synthesis in the field of a powerful flux of high-energy electrons on powder mixtures. The synthesis is carried out via the direct exposure of the radiation flux to a mixture with high speed (up to 10 g/s) and efficiency without the use of any methods or means for stimulation. These synthesis qualities provide the opportunity to optimize compositions and conditions in a short time while maintaining the purity of the ceramics. The possibility of synthesizing ceramics from powders of metal oxides and fluorides (MgF_2_, BaF_2_, WO_3_, Ga_2_O_3_, Al_2_O_3_, Y_2_O_3_, ZrO_2_, MgO) and complex compounds from their stoichiometric mixtures (Y_3_Al_3_O_12_, Y_3_Al_x_Ga_(5−x)_ O_12_, MgAl_2_O_4_, ZnAl_2_O_4_, MgWO_4_, ZnWO_4_, Ba_x_Mg_(2−x)_ F_4_), including activators, is demonstrated. The ceramics synthesized in the field of high-energy electron flux have a structure and luminescence properties similar to those obtained by other methods, such as thermal methods. The results of studying the processes of energy transfer of the electron beam mixture, quantitative assessments of the distribution of absorbed energy, and the dissipation of this energy are presented. The optimal conditions for beam treatment of the mixture during synthesis are determined. It is shown that the efficiency of radiation synthesis of ceramics depends on the particle dispersion of the initial powders. Powders with particle sizes of 1–10 µm, uniform for the synthesis of ceramics of complex compositions, are optimal. A hypothesis is put forward that ionization processes, resulting in the radiolysis of particles and the exchange of elements in the ion–electron plasma, dominate in the formation of new structural phases during radiation synthesis.

## 1. Introduction

Luminophores and scintillators are subject to stringent requirements regarding their response to the influence of the surrounding environment. These materials should not undergo any changes in their properties during the extended operation of the devices in which they are employed, even when operating conditions change. At the same time, they need to be sensitive to ultraviolet radiation and harsh radiation (high-energy particle flux, gamma, and X-ray radiation). Materials based on metal oxides and fluorides are used to achieve high resistance to potential external factors. High sensitivity to UV and harsh radiation is achieved by introducing activators and intrinsic structural defects (luminescent centers) that effectively convert absorbed radiation energy into light, which is detected by light-sensitive receivers.

Optical materials based on metal oxides and fluorides have found wide-ranging applications as phosphors for LEDs [1,2], scintillators [3,4], and long-lasting afterglow markers [5]. These materials are also employed as temperature sensors [6] and in transparent optical media [7]. The ability to visualize radiation flux fields using phosphors and scintillators has led to the development of entire branches of medicine. Effective methods of medical diagnostics, such as X-ray and tomography, are evolving and gaining widespread use [8,9,10]. Each application area requires materials with specific properties. There is a growing demand for the development of new materials with novel characteristics, complex elemental compositions, and structures. Metal oxides and fluorides are promising for use in the mentioned application areas, offering a multitude of material options to meet the increasing practical needs.

The synthesizing of refractory dielectric materials remains a complex task. It requires not only high temperatures but also the creation of new materials from basic elements with significantly different melting temperatures. Therefore, complex multi-step technological techniques are employed for synthesis, creating conditions that promote the exchange of elements in the initial materials through the addition of supplementary substances and mechanical manipulation.

Numerous research studies have been conducted aimed at developing and improving methods for synthesizing materials based on refractory oxides and metal fluorides. In works [11,12,13], a brief description of the synthesis methods employed and their comparison is provided.

The thermal methods have gained the widest acceptance [14,15]. In the thermal synthesis method, high-quality initial materials, typically in the form of finely dispersed powders with a specified stoichiometric composition, are carefully mixed and heated to temperatures below the melting point of the most easily melted component. Over an extended period of time in the heated mixture, partial sintering and element exchange occur. To expedite the process of diffusional element exchange between particles of varying composition in the mixture prior to heating, a flux is added, a substance with a melting temperature lower (approximately 70%) than that of the most easily melted component in the mixture. This serves to replace solid-state diffusion processes with significantly higher rates of liquid-phase diffusion. After cooling, the obtained ceramic is crushed into micrometer-sized particles. Subsequently, multiple high-temperature annealing cycles are conducted (at a temperature of approximately 80% of the melting temperature) for 40–50 h to complete the formation of the desired phase and the evaporation of the flux. Consequently, the process of forming a new phase through thermal methods is time-consuming, and it may be challenging to eliminate all substances introduced during synthesis. Nevertheless, this method is the most widely used and allows for the production of a high-quality final product with high reproducibility due to the careful adherence to technological regulations.

There are also alternative methods of synthesis [16,17,18,19]. For example, the sol–gel method involves the formation of molecules with the desired composition through chemical reactions from precursors. These reactions are carried out in an aqueous solution of nitrates of cations in stoichiometric proportions. Then, a lengthy multi-stage process of removing excess elements follows, along with multiple high-temperature annealing cycles to complete the formation of particle structure and size. The advantage of this method is the ability to obtain powders with a specified particle size distribution. However, the synthesis process is challenging to control and time-consuming.

Exploring the possibility of synthesizing refractory dielectric materials using the combustion method, synthesis in a burner flame [20,21,22] is also considered. The mixture prepared for synthesis is blended with combustible materials and heated to the ignition temperature of the fuel. The desired structure is formed in the high-temperature flame of the combustible. The synthesis time takes only a few minutes, which is the primary advantage of this method. However, post-synthesis cleaning of the obtained powder from residual combustibles is necessary.

In recent years, much attention has been given to the synthesis of refractory ceramics using the spark plasma sintering method [23]. Large currents, representing discharges within the material, are passed through the prepared mixture. The material rapidly melts, leading to the formation of new phases. Substances to enhance conductivity can be added to the mixture to increase current flow. Synthesis can be performed under high pressure and in any atmosphere. The synthesis is completed within minutes, and it is possible to obtain transparent ceramics. This method holds promise, and research is ongoing to explore the most optimal applications.

The influence of intense radiation fluxes during synthesis may promote the necessary solid-state reactions between elements in the environment and enhance the efficiency of new structure formation [24]. Exceeding certain power thresholds in radiation flux can lead to changes in the character of exchange reactions between particles in the environment, with reactions potentially involving short-lived radiolysis products. In [25,26,27], it is shown that high-power, high-energy electron fluxes can be used for synthesizing refractory dielectric materials with high efficiency.

It is established that the synthesis of ceramics from metal oxides and fluorides in the presence of a powerful high-energy electron flux takes less than 1 s, without the use of any synthesis-facilitating substances, and with high productivity. Further research is needed to understand the dependence of synthesis results, the efficiency of new phase formation, their properties, on the technological parameters of radiation synthesis, the history of initial substances, and the limits of universality of the developed method. It is presumed that processes related to the ionization of dielectric materials dominate during ceramic formation. Research into the processes occurring in the mixture under the influence of a powerful ionizing radiation flux is essential.

This work presents a compilation of existing results on the synthesis of luminescent inorganic materials based on metal oxides and fluorides and their analysis, as conducted by the authors up to the present time.

## 2. Ceramic Synthesis

The radiation synthesis method is fundamentally new. At the outset of the synthesis work, the radiation treatment modes, requirements for initial materials, and potential mutual influences between the radiation processing conditions and the properties of the initial materials were completely unknown. Below, we present, describe, and discuss the achieved results of synthesizing a group of ceramic samples differing in their physicochemical properties.

### 2.1. Ceramic Synthesis Methodology

Ceramic synthesis was achieved by directly exposing an electron beam to an initial mixture of a specified composition at the ELV6 electron accelerator at the Budker Institute of Nuclear Physics, Siberian Branch of the Russian Academy of Sciences. High-energy electron beams with energies of 1.4, 2.0, and 2.5 MeV were utilized for the synthesis. The resulting beam, extracted through a differential pumping system, exhibited a Gaussian-shaped profile with an area of 1 cm² on the target surface. Synthesis occurred when the threshold power density of the energy flux was exceeded. Since the penetration depth of electrons into the target increases with their energy, the power density of the beam was adjusted to account for changes in the absorbed energy distribution profile. For the synthesis of yttrium aluminum garnet (YAG) ceramics at 1.4 MeV, the optimal electron beam power densities were in the range of 20–25 kW/cm², while for 2.5 MeV, it was 37 kW/cm² [27].

To synthesize ceramics of the required composition, a mixture of oxide and fluoride powders was prepared in a stoichiometric ratio. To activate the process, oxide powders of rare-earth elements and tungsten were added in quantities ranging from 0.2% to 1.0% of the total mixture mass. Powders of various compositions, prehistory, and with different degrees of dispersion were used. The bulk density (mass of 1 cm^3^ of powder) ranged from 0.2 g/cm^3^ for nanopowders to 2.5 g/cm^3^ for crushed crystals. The mixture was loaded into a massive copper crucible with a surface area of 10 × 5 cm^2^ and depths of 7, 10, and 14 mm. The crucible’s depth for the synthesis of specific ceramics was chosen to ensure the complete absorption of the electron beam of a specified energy by the mixture. The thickness of the mixture layer should exceed the linear range of penetration to prevent the crucible material from entering the forming ceramic. The linear penetration depth (in centimeters) is calculated from the known mass range of penetration σ (g/cm^2^) and bulk density ρ (g/cm^3^) according to the relationship: l = σ/ρ. The crucible was positioned under the accelerator’s exit aperture on a substantial metal table. Two different methods of radiation treatment were used depending on the experimental goals:

1. The electron beam was scanned at a frequency of 50 Hz in the transverse direction of the crucible, while the crucible was moved relative to the scanning beam at a speed of 1 cm/s. This mode is referred to as “R1” in the text.
2. The electron beam was directed at the crucible, which was shifted without scanning at a speed of 1 cm/s relative to the electron beam. This mode is denoted as “R2.”

The total exposure time of the electron beam on the treated surface of the mixture in the crucible was always 10 s due to the design features of the accelerator and the subject table. To achieve equal absorbed doses using both methods, the beam power in the “R2” mode was set to be 5 times lower than in the “R1” mode. Optimal values for the electron beam current densities for synthesizing ceramics from the mixture of selected initial powders were determined experimentally. For all the compositions investigated in this study, the sufficient electron beam power density with an energy of 1.4 MeV fell within the range of 10–25 kW/cm^2^ in the “R1” mode or 2–7 kW/cm^2^ in the “R2” mode. When using electron beams with energies of 2.0 and 2.5 MeV, the beam power density increased by 1.3 and 1.5 times, respectively, to achieve equal absorbed doses [27].

**Figure 1:**
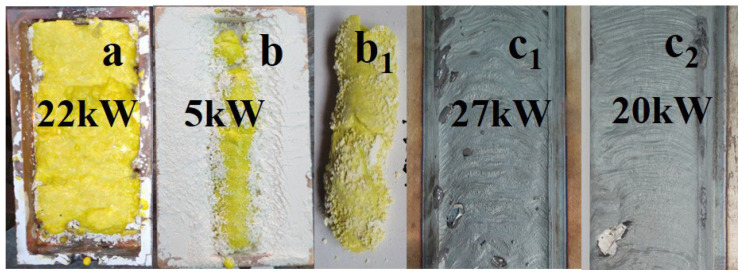
Photographs of YAG:Ce samples synthesized under the influence of an electron beam with E = 1.4 MeV: (**a**) P = 22 kW/cm^2^, R1; b,b_1_ P = 5 kW/cm^2^, R2 in crucibles; (**b**) removed from the crucible; (**b_1_**) traces of the impact of beams with P = 27 and 22 kW/cm^2^, R1 mode, on a steel plate (**c_1_**,**c_2_**).

The synthesis of ceramics was achieved solely through the energy flux of radiation, using only the materials of the mixture, without the addition of other materials facilitating the process. The synthesis resulted in obtaining specimens in the form of plates with dimensions similar to the crucible: 10 × 5 cm² rods, as shown in Figure 1, or a series of specimens in the form of solidified droplets, rods. Samples from the same series, obtained in one experiment in one crucible, have identical structural and luminescent properties. Since the beam had a size in the target plane of 1 cm², each elementary surface area of the mixture was subjected to a 1 s exposure. In mode R2, the flux magnitude in each irradiated elementary area increased and then decreased according to a Gaussian law as the crucible moved relative to the beam. In mode R1, to achieve the same dose of radiation, the integral exposure time remained the same, but the radiation exposure to the elementary surface area of the target was carried out by a series of increasing and decreasing Gaussian-shaped envelope pulses with a duration of 2 ms and a period of 10 ms. Consequently, the synthesis was accomplished in less than 2 ms.

### 2.2. YAG:Ce Ceramics

For the synthesis of YAG:Ce ceramics, a mixture with a stoichiometric composition was prepared, consisting of 57 wt% Y_2_O_3_ and 43 wt% Al_2_O_3_. Cerium oxide (Ce_2_O_3_) was added for activation in an amount ranging from 0.2 to 2 wt% of the total weight. All initial materials had a purity level of no less than 99.5% and were thoroughly mixed. The YAG:Ce ceramic synthesis mixture consisted of Y_2_O_3_, Al_2_O_3_, Ga_2_O_3_ oxides, with 1 wt% Ce_2_O_3_, and a specified Al/Ga ratio. YAG:Ce ceramics were also synthesized with up to 15% substitution of yttrium by gadolinium (Gd). When using an electron beam with E = 1.4 MeV for synthesis in the R1 mode, power density values in the range of 10–25 kW/cm^2^ were employed. A typical appearance of YAG:Ce ceramic plates and rods in the crucible obtained as a result of the synthesis is shown in Figure 1a,b.

When processing with an electron beam at E = 1.4 MeV and P = 22 kW/cm^2^, a ceramic plate with dimensions of 40 × 90 × 7 mm^3^ is formed in the crucible under condition R1. Exposure to an electron beam with E = 1.4 MeV and P = 5 kW/cm^2^ in mode R2 leads to the formation of an YAG ceramic rod inside the mixture with an open upper surface. The rod has a thickness of approximately 5 mm. Often, a series of ceramic samples in the form of solidified droplets are formed in the crucible. The number of samples in one series in a single crucible can vary from one to ten, with sizes ranging from 3 to 30 mm, depending on the properties of the initial materials, irradiation modes, and bulk density. The samples have a solid outer shell with a thickness of up to 2 mm and a porous structure inside. The total weight of a series of samples in the crucible when exposed to an electron beam with E = 1.4 MeV usually ranges from 20 to 30 g. The maximum sample mass obtained under our synthesis conditions at E = 2.5 MeV was 83 g.

In the same figure, a photograph is provided showing traces of the impact on a steel plate (c_1_ and c_2_) by a flux of electrons with E = 1.4 MeV, P = 27, and 20 kW/cm², R1. When exposed to a flux with P = 27 kW/cm², the thin sub-millimeter layer on the surface of the metal plate exhibits melting, whereas at 20 kW/cm², a thin film appears. At lower P values, only an oxidation layer forms on the plate. Thus, the response of the dielectric and metallic materials to radiation treatment is entirely different.

**Figure 2:**
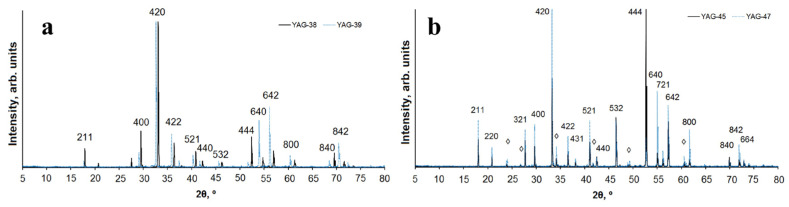
Diffraction patterns of YAG samples: (**a**) 1 (solid line) and 2 (dotted line); (**b**) 3 (solid line) and 4 (dotted line). Reflexes belonging to accompanying phases are marked with the symbol ◊.

The structure of the ceramics was studied using a D8 ADVANCE Bruker diffractometer with a Cu*K*α radiation source. For qualitative phase analysis and diffraction pattern indexing, the following data from the PDF-2 database (ICDD, 2007) were used: PDF 01-089-6659 “Yttrium Gallium Aluminum Oxide (Y_3_Ga_2_Al_3_O_12_),” PDF 00-033-0040 “Aluminum Yttrium Oxide (Al_5_Y_3_O_12_),” PDF 01-070-1677 “Yttrium Aluminium Oxide (YAlO_3_),” and PDF 00-046-1212 “Aluminum Oxide (Al_2_O_3_).” The diffraction patterns of samples from four typical series, namely, YAG: Ce ceramics (1 and 2) consisting of Y_2_O_3_, Al_2_O_3_, Ga_2_O_3_ with 1 wt% Ce_2_O_3_, varying in the Al^3+^/Ga^3+^ ratio; and YAG: Ce ceramics (3,4) with different morphologies of the initial materials, are shown in Figure 2. The presented research results indicate that the dominant structural type for all studied samples is yttrium aluminum garnet (YAG). Samples 1 and 2 are single-phase. Samples 3 and 4 contain yttrium aluminum perovskite (YAP) as a secondary phase with a content of approximately 7% and 11%, respectively.

YAG crystallizes in the cubic symmetry, possesses an elementary I-cell, and belongs to the space group *Ia–3d*. Ce^3+^ ions partially substitute for Y^3+^. In samples 3 and 4, Al^3+^ ions occupy both tetrahedral and octahedral structural positions, whereas in samples 1 and 2, the octahedral position is primarily occupied by Al^3+^/Ga^3+^ cations in approximately a 50/50 ratio, with the tetrahedral position predominantly occupied by Al^3+^ ions in sample 1 and Ga^3+^ ions in sample 2. Similar observations were made in [28].

Due to the difference in ionic radii between Al and Ga [29], the unit cell volume of Y_3_AlGa_4_O_12_ (sample 2) significantly exceeds the volume calculated for the unit cell of Y_3_Al_4_GaO_12_ (sample 1). This is reflected in Figure 2, where the diffraction peaks of sample 2 are shifted towards smaller 2θ angles. Samples 3 and 4 are nearly identical in their phase composition, containing YAG and YAP in approximately the same ratio, with the unit cell parameters for Y_3_Al_5_O_12_ and YAlO_3_ being very close. It can be concluded that the phase composition of the resulting sample depends on the composition and morphology of the initial material.

**Figure 3:**
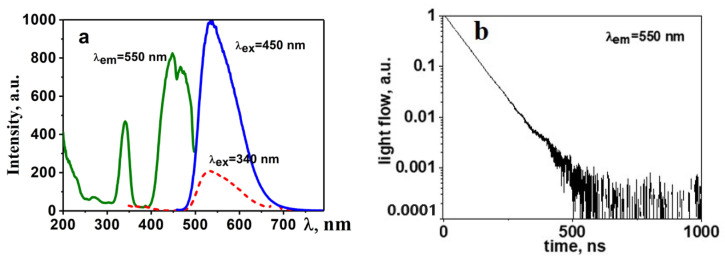
Excitation, luminescence (**a**), and decay kinetics spectra (**b**) of synthesized YAG: Ce ceramics.

A cycle of research on the luminescent properties of synthesized YAG: Ce ceramics has been conducted, including photoluminescence (PL) and cathodoluminescence (CL) spectra under stationary conditions using the Cary Eclipse spectrofluorimeter for excitation and time-resolved cathodoluminescence spectra utilizing a pulsed electron accelerator with an energy of 250 keV [30]. In a generalized form, the spectral–kinetic properties of the obtained YAG: Ce ceramic are depicted in Figure 3b.

Luminescence is efficiently excited by UV radiation in the range of 350 to 450 nm. The maximum of the broad luminescence band occurs at 550–560 nm when excited at λ = 450 nm. In samples of synthesized YAG: Ce ceramics, the luminescence spectrum exhibits typical characteristics of YAG: Ce phosphors with a dominant band around 550 nm and a characteristic relaxation time of 60–65 ns.

As evident from the presented results, the qualitative characteristics of luminescence (spectra, dynamics of their relaxation) are similar to those known for YAG: Ce phosphors [31,32]. In the synthesized YAG: Ce ceramic, crystallites with a structure characteristic of YAG:Ce crystals are formed.

### 2.3. Spinel

For the synthesis of MgAl_2_O_4_ ceramics, a mixture with a stoichiometric composition was prepared, consisting of 28.4% MgO and 71.6% Al_2_O_3_. For the synthesis of ZnAl_2_O_4_ ceramics, the batch comprised 44.4% ZnO and 55.6% Al_2_O_3_. Oxides RE (Eu, Er) were added for activation in an amount of 0.5% by weight. All initial materials had a purity level of not less than 99.5% and were thoroughly mixed.

**Figure 4:**
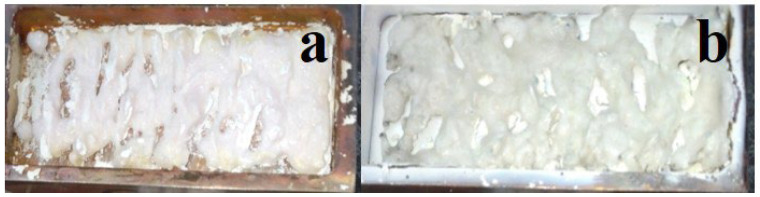
Photographs of MgAl_2_O_4_ (**a**) and ZnAl_2_O_4_ (**b**) sample synthesized at 27 kW/cm^2^.

During the radiation treatment of the mixture in the crucible, a zigzag-shaped ceramic plate forms across the entire area. Examples of the synthesized samples in the crucible are shown in the photographs in Figure 4. The samples resemble solidified melt. The formation of spinel ceramic samples occurs over a wide range of power densities used, ranging from 15 to 25 kW/cm^2^, in the R1 mode. The samples are brittle, and their brittleness decreases with an increase in power density.

**Figure 5:**
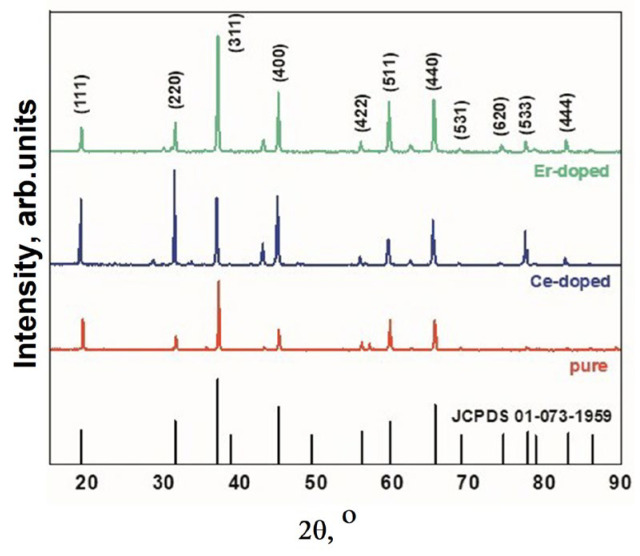
XRD spectra of MgAl_2_O_4_ spinel, pure and doped Ce and Er ions.

The structural properties of MAS were calculated and analyzed using X’pert highscore and Origin software (OriginPro 2018 (64 bit) SR1 b9.5.1.195). Diffractograms of the samples of magnesium aluminate spinel, pure and cerium- and erbium-ion activated, are presented in Figure 5.

XRD results have shown that the synthesized MgAl_2_O_4_ by radiation method have cubic structure and are in the crystalline spinel MgAl_2_O_4_. In addition to the spinel main phase, there are weak peaks of the phase MgO. The weak peaks of phase MgO and no peaks related to Al_2_O_3_ indicate the rather high purity of the obtained MAS. The clear diffraction peaks demonstrate the good crystallinity of the synthesized MAS. The average crystallite size for samples with various impurities is approximately 48 nm. The structural characteristics are in good agreement with known data [33,34].

**Figure 6:**
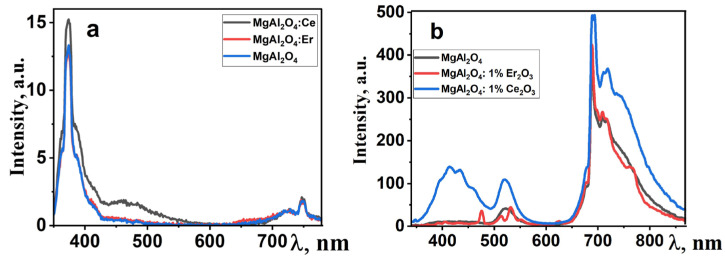
Photoluminescence (**a**) and cathodoluminescence (**b**) images of polycrystalline spinel samples doped with rare-earth elements.

The luminescence properties of ceramic samples were investigated under various excitation conditions. Photoluminescence (PL) under excitation with λ_ex_ = 330 nm was measured using an Agilent Cary Eclipse spectrofluorimeter, while cathodoluminescence (CL) excitation was achieved using a pulsed electron accelerator with an energy of 250 keV [35]. The spectral characteristics of the synthesized MAS luminescence are presented in Figure 6.

In the photoluminescence spectra (Figure 6a), an intense emission band is observed in the UV region, along with broad emission in the “red” region of the spectrum. Under electronic excitation, the dominant emission in the spectrum is the “red” emission, both for pure and activated spinel samples. Additionally, there are bands with maxima at around 410 and 520 nm, and their intensity depends on the type of doping impurity. The emission in the region with λ_max_ = 380 nm may be associated with F-centers in the spinel structure. The emission band in the 700–760 nm range is attributed to oxygen vacancies. A characteristic feature of spinel is the emission of chromium ions, which appears as a set of narrow bands in the 700 nm region [36]. As evident from the presented results, the qualitative characteristics of luminescence are similar to well-known ones [37,38]. In this manner, in the synthesized MgAl_2_O_4_ ceramic, crystallites with a spinel-like structure are formed.

### 2.4. Fluorides

Fluorides of alkaline earth metals (MeF_2_) and their solid solutions have been utilized as scintillation materials [39,40,41]. The synthesis of ceramics based on alkaline earth metal fluorides (MeF_2_) is of interest for several reasons. The activation of crystals by multivalent ions makes them sensitive to the effects of ionizing radiation. However, introducing the most interesting ions for increasing sensitivity, such as W and U, is hindered by the fact that in a fluoride environment at temperatures of 20–60 °C, these ions form volatile compounds and are removed from the medium. Therefore, additives capable of retaining W and U ions in the melt are introduced into the crucible for the synthesis of such compounds. Incorporating hexavalent ions W and U into the lattice is complicated by the need to balance the charge difference, which can be resolved by using solid solution matrices, such as BaMgF_4_. Finally, the melting temperatures of MeF_2_ are 1.5–2 times lower than those of metal oxides. This is of interest in understanding synthesis processes in a radiation field. It can be expected that at a high rate of radiation synthesis, the activator ions will not have time to exit the ceramic formation zone [42].

Activated W ceramic samples of BaF_2_, MgF_2_, and BaMgF_4_ were synthesized. For the ceramic synthesis, tungsten oxide was added to the mixture in amounts of 1–2 weight percent for activation. All initial materials had a purity level of no less than 99.5% and were thoroughly mixed.

**Figure 7:**
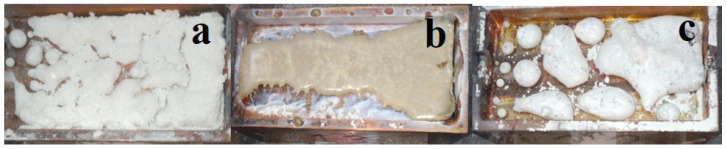
Photographs of activated W samples of BaF_2_ (**a**), MgF_2_ (**b**), and BaMgF_4_ (**c**) ceramics synthesized under the influence of an electron beam with E = 1.4 MeV, P = 15 kW/cm^2^, R1.

When using an electron beam with E = 1.4 MeV under the R1 mode, power densities in the range of 10–20 kW/cm^2^ were employed for synthesis. A typical appearance of activated W ceramic samples of BaF_2_, MgF_2_, and BaMgF_4_ synthesized under the influence of an electron beam with E = 1.4 MeV and P = 15 kW/cm^2^ in the R1 mode is shown in Figure 7.

During the radiation treatment of the mixture in the crucible, ceramic samples solidify in the form of a melt. Ceramic formation occurs over a wide range of used power densities from 10 to 20 kW/cm^2^, in the R1 mode.

The results of phase analysis of the diffractograms for the synthesized fluoride samples of Ba and Mg using a power density of P = 15 kW/cm^2^ are presented in Table 1.

The synthesis result depends on irradiation modes and the history of the initial substances. With changes in power density, the phase composition of the synthesized samples can undergo some variations: in MgF_2_, MgO phase is detected, and in BaMgF_4_, BaF_2_ is preserved. The appearance of the MgO phase can be explained by oxidation: the synthesis is conducted in an open atmosphere. Nevertheless, XRD results show that during radiation treatment, ceramics with the mentioned phases, including complex ones, are formed.

**Figure 8:**
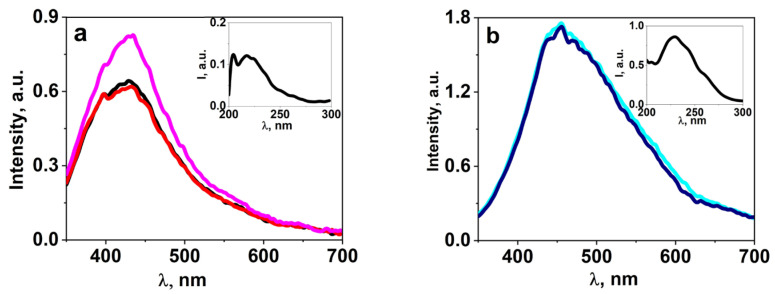

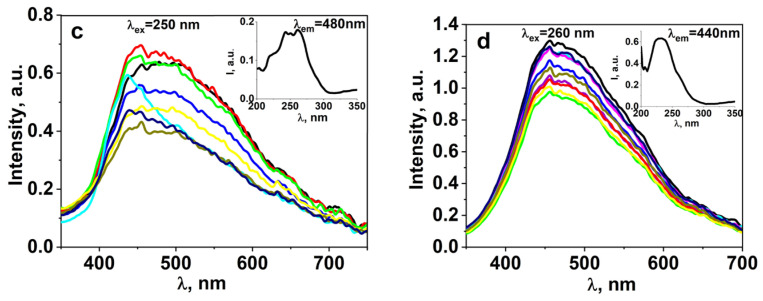
The FL spectra of BaMgF_4_ (**a**) samples under excitation at 220 nm, as well as BaMgF_4_:W (**b**), BaF_2_:WO_3_ (**c**), and MgF_2_:WO_3_ (**d**) samples under excitation in the 260 nm range.

A series of studies on the luminescent properties of W-activated samples of synthesized ceramics BaF_2_, MgF_2_, BaMgF_4_ was carried out: photoluminescence (PL) excitation and emission spectra in stationary conditions using the SM2203 SOLAR spectrometer. In a generalized form, the spectra are presented in Figure 8. The figures present the results of spectral measurements of fluorescence from individual sections of the sample to demonstrate the homogeneity of properties. The inserts in the spectra show the excitation spectra of luminescence. The spectra highlighted in different colors in the graph (Figure 8) indicate that the spectra of the same sample from different regions are highlighted in different colors.

Research on the spectral properties of synthesized ceramic samples has revealed two significant differences between activated and non-activated samples. In activated ceramic samples, the position of the luminescence band is shifted towards the longer-wavelength region. In non-activated samples, luminescence is excited by UV radiation up to 260 nm, while in activated samples, it extends up to 300 nm.

**Figure 9:**
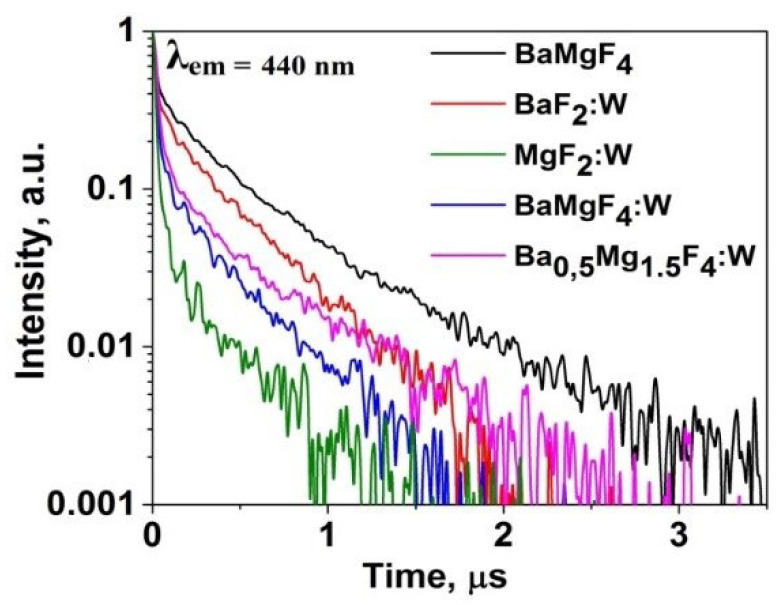
Kinetic decay curves of CL in ceramic samples.

Kinetic decay curves were measured following excitation of cathodoluminescence using electron beam pulses with an energy of 250 keV and a duration of 10 ns. The measurement results are presented in Figure 9. In all activated ceramic samples, the appearance of a short-lived component is observed.

The combination of the obtained research results on synthesized ceramic samples based on alkaline earth metal fluorides allows us to draw the following conclusion: during synthesis under the influence of intense radiation fluxes, tungsten can be successfully incorporated into the ceramic lattice. Within a short synthesis period, tungsten does not have sufficient time to exit the synthesis zone and remains trapped within the lattice.

### 2.5. Tungstates

Tungstates (MeWO_4_, Me: Mg, Ca, Cd, Zn) are promising materials for use as scintillation materials [43,44,45,46]. To advance our understanding of the physicochemical processes during radiation synthesis in dielectric materials, tungstates are of interest due to the necessity to synthesize them from a mixture with significantly different melting temperatures: WO_3_ (1473 °C), ZnO (1975 °C), MgO (2570 °C), CaO (2572 °C), CdO (1559 °C).

**Figure 10:**
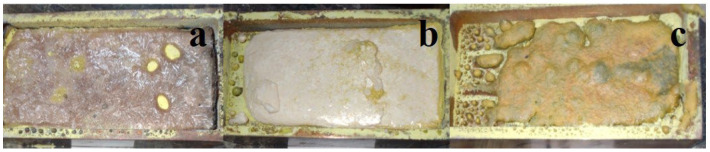
Photographs of ceramic samples (**a**) ZnWO_4_, (**b**) MgWO_4_, (**c**) CaWO_4_, synthesized under the influence of an electron beam with E = 1.4 MeV, P = 18 kW/cm^2^, R1.

We have successfully synthesized ceramic samples of MeWO_4_, where Me represents Mg, Zn, Ca, from the following mixture compositions: MgWO_4_ (MgO 14.7%, WO_3_ 85.3%); ZnWO_4_ (ZnO 26%, WO_3_ 74%); CaWO_4_ (CaO 19.5%, WO_3_ 80.5%). After mixing, the mixture was poured into a crucible and compacted to even out the surface. All initial materials had a purity level of no less than 99.5% and were thoroughly mixed. For the synthesis, electron irradiation with E = 1.4 MeV at power densities ranging from 10 to 25 kW/cm^2^ in R1 mode was employed. The typical appearance of the synthesized ceramic samples after exposure to an electron beam with E = 1.4 MeV, P = 18 kW/cm^2^, R1 mode, is shown in Figure 10.

In the process of synthesis, ceramic samples were formed in the shape of plates with dimensions similar to that of a crucible. It should be emphasized that ceramic formation occurs from a mixture composed of initial metal oxides with significantly different melting temperatures under identical conditions of radiation treatment.

The surface structure of the synthesized samples of ZnWO_4_, MgWO_4_, and CaWO_4_ tungstates was investigated using a scanning electron microscope, Mira 3 (TESCAN). Since the examined samples are dielectrics, they were coated with a conductive carbon layer using a Quorum Q150R ES sputtering system. The investigation was conducted at an accelerating voltage of 25 kV.

**Figure 11:**
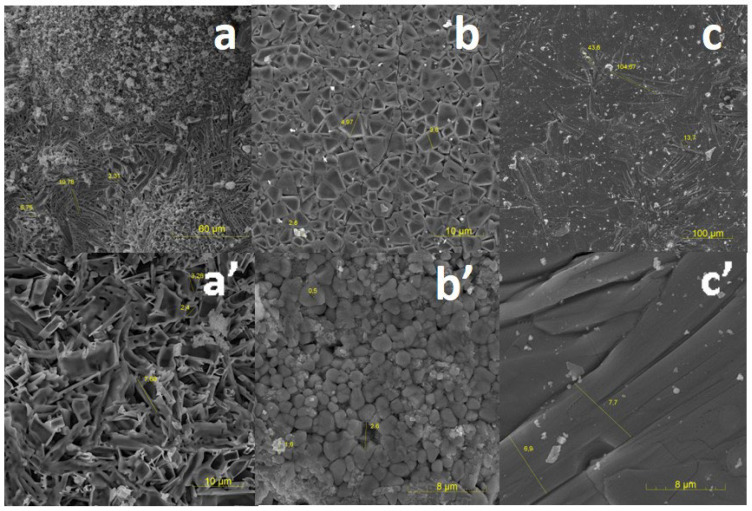
SEM images of the surface of ceramic samples (**a**,**a′**)ZnWO_4_, (**b**,**b′**) MgWO_4_, and (**c**,**c′**) CaWO_4_ synthesized under the influence of an electron beam with E = 1.4 MeV, P = 18 kW/cm^2^, R1.

SEM images of the measured samples are presented in Figure 11. On the surface of the ZnWO_4_ samples, large formations of spherical shape with dimensions on the order of 180 µm, which can reach sizes of up to 600 µm, are visible. Upon increasing the image resolution, a porous microstructure with elongated elements ranging in size from 7 to 20 µm and a thickness of approximately 7 µm is observed. These elements may be microcrystals of the synthesized material.

On the surface of the MgWO_4_ sample, a dense compact microstructure with inclusions (approximately 50 µm) and small holes (10–20 µm) is observed. With an increase in image resolution, tightly packed microcrystals of various shapes with average sizes ranging from 2 to 5 µm become visible.

On the surface of the CaWO_4_ sample, densely packed microcrystals of elongated shape with an average length of 100 µm and a width of 7 µm are observed.

The presented results suggest the possibility of forming a crystalline structure in the synthesized samples.

### 2.6. Initial Materials for the Synthesis of Metal Oxides and Fluorides

The necessity of establishing the possibility of synthesizing ceramics from the materials used for the synthesis of the aforementioned functional materials is entirely evident. We conducted experiments on ceramic synthesis using powders of all the oxides and fluorides of the following metals: Y_2_O_3_, Al_2_O_3_, Ga_2_O_3_, MgO, WO_3_, ZnO, ZrO_2_, MgF_2_, BaF_2_. To synthesize the powders, they were placed in crucibles, and the conditions of radiation treatment were experimentally selected, including the mode and power density. Additionally, experiments were conducted for the synthesis of activated ceramics, where an activator in the form of its oxide was added to the powder. The introduction of the activator allowed us to demonstrate its potential incorporation into the crystalline lattice through luminescence techniques.

**Figure 12:**
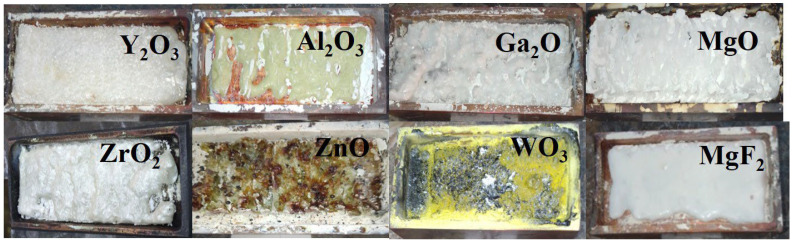
Photographs of synthesized ceramic samples under the influence of a 1.4 MeV electron beam in mode R1: Y_2_O_3_; Al_2_O_3_; MgO; ZrO_2_ (P = 25 kW); ZnO (P = 22 kW); Ga_2_O_3_ (P = 18 kW); WO_3_ (P = 17 kW); MgF_2_ (P = 15 kW).

In Figure 12, photographs of ceramic samples made from the primary types of substances used in the synthesis of functional materials are presented. It was found that the synthesis results, primarily the efficiency of converting initial materials into ceramics, strongly depend on their prior history. We understand the prehistory not only in terms of the quality of the chemical composition but also in terms of dispersity, the sizes and quantity of particles, and their distribution by size. The available information about the obtained materials was insufficient for selection. Therefore, substances with different histories were used for synthesis. Figure 12 presents the results of the radiation synthesis of ceramic samples from the initial substances that provided the highest efficiency. More details about the dependence of synthesis efficiency on the history of the initial materials will be discussed below.

Among all the selected initial substances used for synthesis, except for WO_3_, it was possible to obtain ceramic plates with dimensions comparable to the crucible sizes. Introducing additives into the mixture to obtain activated samples in the required quantity up to 2 wt% does not affect the synthesis results. The conditions of radiation treatment for ceramic synthesis, particularly power density, were primarily selected through experimental means.

One can draw the following conclusion from the above:

A series of experiments has been conducted to investigate the possibility of synthesizing ceramics based on oxides and metal fluorides by direct exposure to high-energy electron beams on a stoichiometric mixture.

It has been determined that under the influence of electron beams with energies in the range of E = 1.4–2.5 MeV and power densities of P ~10–25 kW/cm^2^, it is possible to form ceramics, including activated ceramics, with a crystalline structure of YAG (Y_3_Al_5_O_12_, Y_3_Al_x_Ga_5−x_O_12_), spinels (MgAl_2_O_4_, ZnAl_2_O_4_), and metal fluorides (BaF_2_, MgF_2_, BaMgF_4_). The synthesis of tungstates MgWO_4_ and ZnWO_4_ is also achievable under these conditions.

Under the given radiation exposure conditions, the synthesis of samples occurs at a rate of 1 cm^2^/s. The synthesis time for an elementary section of ceramics from the mixture does not exceed 2 ms.

The synthesis of ceramics is achieved without the use of any additional substances that facilitate the process. Radiation exposure promotes efficient mixing of the mixture components.

During radiation synthesis, it is possible to introduce ions of activators and modifiers into the lattice, which is difficult to achieve using other methods. At high synthesis rates, ions do not have time to leave the reaction zone.

The combination of processes in the mixture during radiation synthesis differs significantly from those initiated by heating during thermal treatments. The synthesis of ceramics from the investigated dielectric materials, which have different melting temperatures, occurs within similar ranges of the applied conditions. The results of radiation exposure in these materials are significantly different from those in metals.

## 3. The Dependence of Radiation Synthesis Efficiency on the Prior History of the Initial Materials

Establishing the relationship between the efficiency of material synthesis and the state and properties of the initial substances is a multifactorial task. Therefore, these studies receive significant attention. Such research is ongoing because the technologies for obtaining initial materials are constantly improving, and their scope is expanding.

It is evident that the requirements for the initial materials vary depending on the use of different technologies. For instance, in thermal synthesis, the efficiency of synthesis (time, quality) is higher when the precursor particle sizes are smaller. Smaller particle sizes increase the likelihood of element exchange between particles. In radiative synthesis, the formation of new structures occurs in the electron-ion plasma created by a powerful stream of high-energy radiation. Therefore, it is necessary to investigate the dependence of the results of radiative synthesis on the properties of the initial substances. In [28], when studying the dependence of the efficiency of radiation synthesis on the history of the initial material, it was found that the synthesis outcome is significantly influenced by the dispersed composition of the substances used for synthesis. This dependence has a greater impact on the synthesis outcome than the degree of their purity. We conducted a series of studies aimed at investigating the dependence of the efficiency of radiation synthesis of ceramics based on metal oxides and fluorides on the particle sizes of the initial powders.

### 3.1. Experimental Assessment of Synthesis Efficiency

To discuss the dependence of synthesis results on the history of powders and radiation treatment conditions, the concept of “synthesis efficiency” is proposed to be introduced. By “synthesis efficiency” here and in the following context, we mean the ratio of the mass of the obtained series of samples in one crucible in a single experiment to the mass of the mixture in the crucible. It should be emphasized that this assessment provides fairly valuable information for establishing the relationship between the synthesis outcome and the conditions of radiation exposure and the history of the initial materials. However, it is important to remember that the obtained samples may be coated with remnants of the mixture in the crucible, which can sometimes be challenging to remove, especially when using the R2 mode. A layer of mixture thicker than the penetration depth of electrons is always added to the crucible to ensure that the crucible material does not affect the results. Finally, a portion of the mixture is dispersed in the radiation field due to the charging of dielectric particles and rapid heating of the air in the mixture. Therefore, the values of the synthesis reaction yield should be considered as approximate. Nevertheless, they provide a good understanding of the efficiency of synthesis of different materials and its dependence on the properties of the initial materials and radiation treatment modes.

Table 2 presents examples of the results of evaluating the efficiency of radiation synthesis of ceramics. To date, a total of 538 experiments have been conducted for the synthesis of ceramics with various compositions and different initial materials. The table primarily showcases the evaluation results of compositions with the highest synthesis efficiency. It is evident that these results can be achieved through radiation synthesis. To provide a comparison, the evaluation results of synthesis with low values are also included to establish the causes of the influence of the precursor’s history.

As noted previously, the impact of a high-energy electron beam on the mixture in the crucible results in the formation of a series of samples ranging from one (yielding a plate) to 10–15. All samples within a given series exhibit similar properties [47], with differences in synthesis efficiency not exceeding 10%. It is worth noting that synthesizing ceramics from a mixture of the same composition and powders with the same history, under identical conditions of radiation treatment, also leads to the formation of specimens with similar properties and an efficiency difference not exceeding 20%. The text and tables include serial numbers for the samples according to the system used by the authors to track synthesis results.

As indicated by the results presented in Table 2, there is a significant dispersion in the values of reaction yield and mass loss. For instance, the synthesis reaction yields of MgO (2), Al_2_O_3_ (nano), and ZrO_2_ (2) ceramics range from 0% to 5%. Meanwhile, under similar radiation exposure conditions, the synthesis reaction yields for MgO (K11) are 90.9%, Al_2_O_3_ (F-800) is 94.9%, and ZrO_2_ (1) is 80%. Based on the available information, these two groups of materials differ in the dispersion of the initial substances used for synthesis.

**Figure 13:**
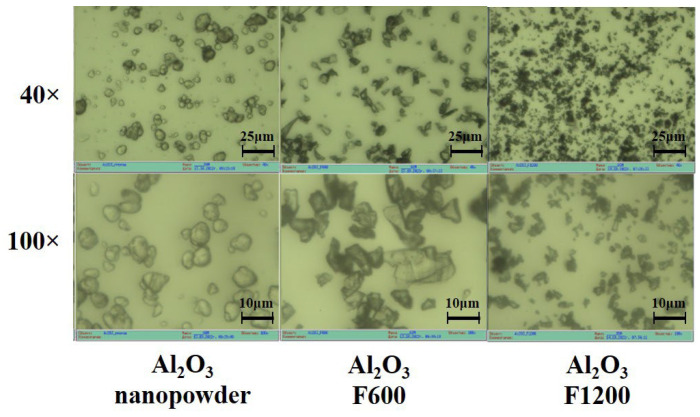
Microphotographs of the initial aluminum oxide powders on zoom.

We conducted studies on the morphology of Y_2_O_3_ powders of grades ITO/I, ITO/B, and Al_2_O_3_ grades F600–F1200, as well as nanooxide, using an optical microscope µVizo (LOMO). Microphotographs of the initial aluminum oxide powders are presented in Figure 13.

The morphology of powders significantly varies. Nanopowder particles exhibit a clearly non-crystalline appearance, typical of agglomerated nanoparticles. The particles of F800 and F1200 powders appear as fragmented crystals with distinct fractures. The sizes of agglomerated nanoparticles and microcrystals are comparable. Therefore, radiation synthesis using nanoparticles is inefficient. A high synthesis efficiency is achieved when particles with sizes of 5–10 µm are used. It is worth noting that there is no such difference in thermal synthesis. The low efficiency of radiation synthesis may be attributed to significant differences in the processes of electronic excitation decay in bulk and nanocrystals. In nanocrystals, radiation-induced electronic excitations localize and decay on the surfaces of nanoparticles without producing radiolysis products.

It is hypothesized that synthesis efficiency is also low for powders of larger sizes. As indicated in the table, the synthesis of ceramics from MgO (G) powder is extremely low. Unfortunately, there is no information about the particle sizes for this material, but direct observations using an optical microscope have shown the presence of a large number of large, sub-millimeter-sized particles. Therefore, to establish the relationship between synthesis efficiency, one needs to know not only the particle sizes but also the full spectrum of particle sizes.

### 3.2. Dispersion of Initial Materials

To establish the validity of this assumption, we conducted a series of measurements of the dispersion of all currently used initial materials. Measurements were performed using the laser diffraction method with a laser particle size analyzer Shimadzu SALD-7101. Below, we will consider the results of the dispersion study of the most commonly used materials in the synthesis of MgO, Al_2_O_3_, Y_2_O_3_ powders, and the possible influence of dispersion on the radiation synthesis of ceramics.

**Figure 14:**
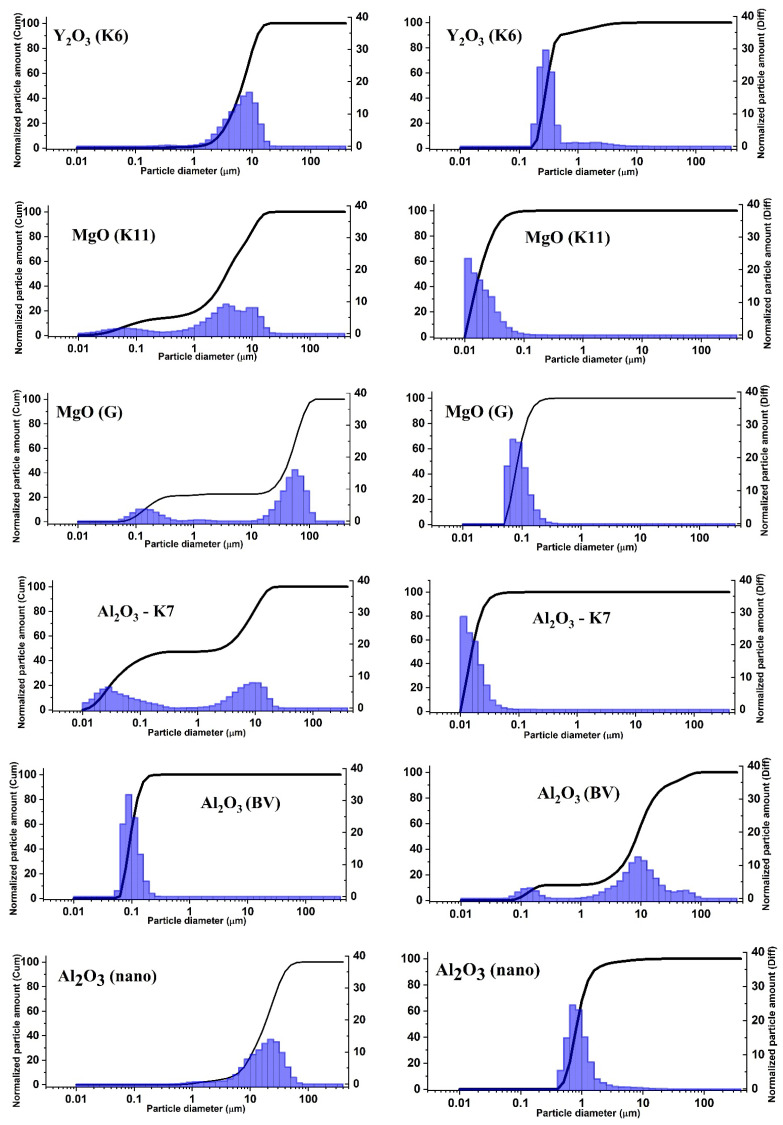
Dependence of the quantity of particles and volume on their sizes in the initial powders of MgO, Al_2_O_3_, and Y_2_O_3_. (**Left**) dispersion by volume; (**right**) dispersion by quantity of particles.

Figure 14 shows the results of the dispersion analysis of MgO, Al_2_O_3_, and Y_2_O_3_ powders with different histories. The method used allows us to obtain relationships between the quantity of particles and their volume as a function of particle size. This may provide the opportunity to establish the nature of processes that determine the relationship between radiation efficiency and particle sizes and optimize the synthesis process by selecting the initial materials.

As can be seen from the presented measurement results of particle distribution, in all examined powders, the distribution of particles by size in terms of volume and quantity does not match. The volume of larger particles is usually much greater than the volume of smaller particles. This conclusion is based not only on the data shown in the figure but also on all 24 measured powders of MgO, Al_2_O_3_, Y_2_O_3_ with different histories. As seen from Table 2, the sample’s mass is almost equal to the mass of the mixture. Therefore, synthesis primarily occurs from the larger particles in the mixture. It has been shown earlier that the efficiency of radiation synthesis is low for nanoparticles. Thus, there exists an optimal range of particle sizes for radiation synthesis, within which the formation of ceramics is most efficient. It is evident that radiolysis of larger particles is less probable than that of smaller ones.

Ceramics synthesis of the listed complex compositions from mixtures of MgO, Al_2_O_3_, Ga_2_O_3_, Y_2_O_3_, ZnO, WO_3_, BaF_2_, and MgF_2_ has been realized. As expected, the efficiency of ceramics synthesis of yttrium aluminum garnets, spinels, tungstates, and alkali earth metal fluorides (BaMgF_4_) depends on the dispersity of the initial powders in the mixture. The ceramics selected here are wide-bandgap dielectrics with a high degree of ionic bonding, where the process of electronic excitation decay into pairs of defects is efficient. The exit of decay products beyond the particle depends on its size. Large ceramic samples with sizes up to 10 cm^2^ and thicknesses of 0.6–0.8 cm are obtained successfully from mixtures of powders with grain sizes of 1–10 µm. Mixtures of powders with different sizes (nano and microparticles) result in smaller samples with significantly lower yields. For example, the efficiency of Y_3_Al_5_O_12_:Ce ceramics synthesis from a mixture of Al_2_O_3_ (K7) and Y_2_O_3_ (K6) is 99.1%, whereas from a mixture of Al_2_O_3_ (nano) and Y_2_O_3_ (ITO-V), the efficiency is 53%. It should be noted that the efficiency of synthesis of the same ceramics from a mixture of Al_2_O3 (BV) and Y_2_O_3_ (ITO-V) does not exceed 50%. In this mixture, the Al_2_O_3_ (BV) powder consists of particles with sizes of 10–50 µm (Figure 14). Thus, the efficiency of Y_3_Al_5_O_12_:Ce ceramics synthesis is maximum when using mixtures of powders with similar dispersion.

A similar conclusion can be drawn from the comparison of the dispersion of initial powders for the synthesis of MgAl_2_O_4_:Eu ceramics. The synthesis was performed using two variants of the initial mixture. Aluminum oxides of grades K7 and F-800 have very similar particle size distributions. The main volume consists of Al_2_O_3_ particles with sizes in the range of 3–12 µm. The sizes of MgO (K11) particles are in the range of 1–15 µm, and Al_2_O_3_ (K7) particles are in the range of 3–12 µm. The efficiency of MgAl_2_O_4_:Eu ceramics synthesis is 99%. From a mixture of Al_2_O_3_ (F-800) powders with particle sizes ranging from 3 to 12 µm and MgO (1) powders with particle sizes ranging from 0.4 to 2 µm, the synthesis occurs with an efficiency of 23%.

The reason for the dependence of the efficiency of ceramics synthesis of complex compositions on particle sizes is the difference in the dispersion of the initial compositions’ powders. When their sizes are unequal, local non-stoichiometry may occur because large particles are surrounded by many small ones. Effective element exchange between such particles cannot occur in a short time. As seen from Table 2 and Figure 14, the synthesis efficiency is optimal when the dispersion of the mixture components is close.

It is worth noting that efficient radiation synthesis of three-component compositions is also possible. A high-efficiency (99.3%) synthesis of ZnAlGaO_4_ ceramics was obtained from a mixture of ZnO, Al_2_O_3_, and G_2_O_3_ powders.

This work does not consider the possible dependence of synthesis efficiency on the purity of the initial substances. As demonstrated by the results of activated ceramics synthesis, introducing activator oxides into the mixture up to concentrations of 1% does not affect efficiency. Apparently, other possible impurities do not influence the synthesis results as much as the difference in dispersion does.

## 4. Energy Losses of an Electron Beam in a Material

In the modes of radiation treatment used for ceramic synthesis, the electron beam interacts with the mixture for a short duration. The beam has a Gaussian distribution in cross-section. Ceramic synthesis was carried out using electron beams of varying energy and power in two different modes: R1 (with scanning) and R2 (without scanning). The absorption of energy by the incident electrons in the material is non-uniform in depth. All of these characteristics of radiation treatment can influence the synthesis outcome. Therefore, a thorough analysis of the energy transfer processes from the electron beam to the material is required.

The energy loss of electrons with energies ranging from 0.5 to 5 MeV when passing through matter predominantly occurs due to ionization processes. The fraction of radiation losses is negligible. The mass depth of run of electrons, in close agreement with experimentally measured values, is calculated using empirical formulae [47,48]. With high precision, it can be calculated based on known values of substance density, average number of electrons in atoms, and the average ionization potential. Modeling the passage of electrons through a substance using the Monte Carlo method allows not only for determining the distribution of energy losses along the path of electron propagation; it also enables a rigorous estimation of energy loss distribution in the transverse cross-section relative to the beam propagation. Such estimations are important in cases where beams of limited cross-section are used, for example, of Gaussian shape. It is precisely such beams that were applied in the current study for ceramic synthesis. The high reliability and quantitative correspondence to experimental results are ensured by Monte Carlo calculations using the CASINO program [49,50,51].

The distribution of absorbed energy in the material when exposed to a non-uniform cross-sectional high-energy electron beam can be accurately calculated using the CASINO V2.5 program.

### 4.1. Energy Loss Modeling

**Figure 15:**
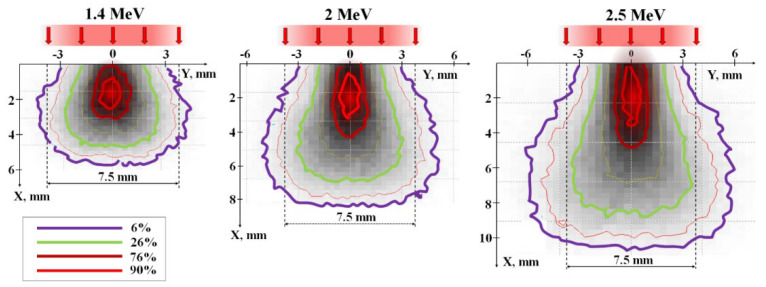
Energy loss distribution of electrons with E = 1.4, 2.0, 2.5 MeV in a mixture with a bulk density of 1.2 g/cm^3^ for the synthesis of Y_3_Al_5_O_12_ ceramics. Colored lines of equal losses are presented in units relative to the losses at the center.

Figure 15 shows profiles of energy loss distribution for electrons passing through a mixture with a bulk density of 1.2 g/cm³ made of Y_2_O_3_ (57%) and A_2_O_3_ (43%) powders, which were used for synthesizing Y_3_Al_5_O_12_ ceramics. The calculations were performed using the Monte Carlo method for beams with Gaussian flux distribution across the cross-section and a total of 10,000 incident electrons at energies of 1.4, 2.0, and 2.5 MeV, which were utilized in the experiments.

When an incoming beam of electrons penetrates a material, it scatters off atoms and ions within the substance, transferring its energy to ionization and the generation of secondary electrons. As a result of these processes, the spatial structure of energy transfer within the beam changes as it progresses through the material. A portion of the energy is imparted to the material beyond the cross-section of the beam. Energy losses are concentrated along the axis of the beam. Approximately 50% of the total energy loss of the beam occurs within the region along the beam axis, with a cross-section of 0.3–0.4 relative to the beam’s surface area. This results in a characteristic distribution of energy losses along the beam axis.

**Figure 16:**
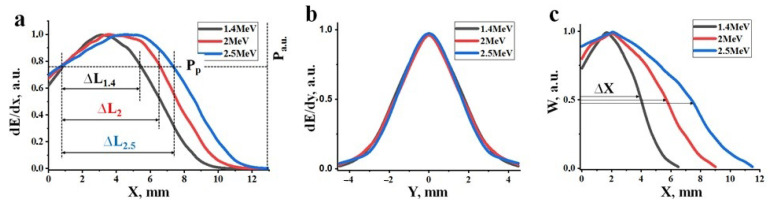
Profiles of energy loss distributions dE/dx (**a**), dE/dy (**b**) for electrons with energies of 1.4, 2.0, 2.5 MeV in a target, and the absorbed energy density W (**c**).

Figure 16a shows calculated profiles of energy loss distribution (dE/dx) of electrons within the material as a function of depth, assuming an equal number of incident electrons with energies of 1.4, 2.0, and 2.5 MeV. Here, energy losses of the electron beam as it passes through the material are understood as the magnitude of losses at depth X across the entire region perpendicular to the beam axis. The maximum absorbed energy is found at a certain depth from the surface, depending on the electron energies. The positions of the peaks in the dE/dx energy loss for the beams correspond to 2.8, 3.7, and 4.6 mm for the specified electron energies. The magnitude of energy losses at the peaks is 30–40% higher than at the surface of the target.

Figure 16b presents calculated profiles of the energy loss distribution of electrons (dE/dy) in the direction perpendicular to the beam axis. From the results presented in Figure 15, it is evident that the profiles of dE/dy change with depth. These changes are different for electron beams of different energies. The profiles of dE/dy shown in the figure correspond to depths that correspond to the maximum density of absorbed energy (Wr) of the electron beam. Here, the absorbed energy density (W) is defined as the energy loss per unit volume of material. The dE/dy profiles coincide, indicating that under the calculation conditions with an equal number of incident electrons, the values of Wr are the same.

Energy loss densities along the axis of the passing beam are always higher than off-axis and exhibit a curve with a maximum. Figure 16c shows calculated profiles of the dependence of W on the depth of electron penetration into a material with a specified composition and bulk density. These dependencies appear as curves with maxima at 1.8, 2.1, and 2.3 mm for electrons with energies of 1.4, 2.0, and 2.5 MeV, respectively. The length of the region (Δx) with equal absorbed energy density along the beam axis increases by a factor of 2 on average as the electron energy increases from 1.4 to 2.5 MeV. It is worth noting that the maximum of energy loss (dE/dx) occur at depths of 2.8, 3.7, and 4.6 mm for energies E equal to 1.4, 2.0, and 2.5 MeV, respectively. However, the length of the region with equal absorbed energy along the beam axis increases by only 25% on average. This is due to the expansion of the energy loss region towards the end of the range. Consequently, the maximum of energy loss with depth (Figure 16a) (dE/dx) and energy loss densities (W) (Figure 16c) do not coincide.

Clearly, regions of maximum energy loss densities should govern subsequent processes. It is within these regions that ionization density is maximized, and the material reaches its highest temperature. In these regions, when the energy loss exceeds a certain threshold, the crystalline structure of yttrium and aluminum oxides transforms into yttrium aluminum garnet. Primarily, synthesis should take place at depths corresponding to the maximum energy loss densities along the beam axis, not just energy loss.

With changes in the power of the electron beam (P), the distribution profile remains unchanged, but the absolute values of energy losses (dE/dx and dE/dy) change proportionally. Synthesis occurs in the material when the energy losses (dE/dx and dE/dy) in a specific region of the material with coordinates X-Y exceed a certain threshold power (Pp) of the beam. The threshold (Pp) at which synthesis can occur depends solely on the composition of the initial mixture, i.e., the composition of the synthesized material. Synthesis may not occur at the surface of the target and at great depths but rather within a depth range (ΔL) where energy losses exceed the required threshold for synthesis. The depth range (ΔL) where synthesis can occur increases with increasing electron energy (E). Synthesis takes place within depths where energy loss densities (W) exceed the required threshold for synthesis. As the power of the electron beam (P) increases, the length and diameter of the region with maximum energy losses (W) along the beam axis increases. Therefore, synthesis can occur in a larger volume where energy loss densities exceed the synthesis threshold (Pp).

In summary, the volumetric energy loss density undergoes significant changes in both the longitudinal and transverse directions to the electron beam propagation in the material. As the power density of the incident electron beam increases, energy losses proportionally increase. In regions of maximum energy loss density, material synthesis is most likely to occur. With higher electron beam power, the volume in which synthesis can occur increases, with the upper limit potentially reaching the surface of the target and the lower limit extending to depths equal to the extrapolated electron range (X_e_). The extrapolated range depth is defined as the value on the depth-of-penetration axis (X_e_) at which the axis intersects the tangent to the energy loss curve at its descent at the inflection point of the function. The extrapolated electron range in the mixturet for YAG:Ce ceramic synthesis (Figure 15) is 9, 10, and 11 mm for electrons with energies of 1.4, 2.0, and 2.5 MeV, respectively.

### 4.2. Experimental Verification of Energy Loss Distribution

A research cycle was conducted to investigate the dependence of the efficiency of YAG:Ce ceramic radiation synthesis on electron energy and beam power. The concentration of the Ce activator introduced for activation was 0.5%. Such a quantity of activator does not affect the fundamental energy loss patterns but allows confirmation through luminescent methods that Ce is incorporated into the ceramic’s crystalline structure. YAG:Ce ceramic synthesis was performed in copper crucibles with depths of 10 or 14 mm, exceeding the full electron range from 1.4 to 2.5 MeV, and dimensions of 50 × 100 mm^2^. The ceramic synthesis was carried out in R1 and R2 modes.

Since the distribution of absorbed energy by the electron beam in the material is non-uniform, it is necessary to define criteria for selecting irradiation conditions for a correct comparison of synthesis results using electron beams with different energies. The maximum energy loss densities in the irradiated material region should be similar. Our previous studies have shown that when using an electron beam with E = 1.4 MeV, the synthesis of YAG:Ce ceramics in R2 mode is successful when delivering 4 kJ/s·cm³ of energy to the central region with a bulk density of 1.2 g/cm³, which corresponds to 50% of the absorbed energy. Such energy absorption density is achieved with a beam power of 5 kW/cm² under the used irradiation conditions. When irradiated with electrons of higher energies, 50% of the absorbed energy occurs in a larger volume along the beam path. Based on the study of the dependence of absorbed energy distribution on electron energy (Figure 15 and Figure 16), we have shown that the electron beam power should be 1.4 times higher for electrons with E = 2.0 MeV and 1.8 times higher for electrons with E = 2.5 MeV. The adjustment of synthesis modes was carried out experimentally.

**Figure 17:**
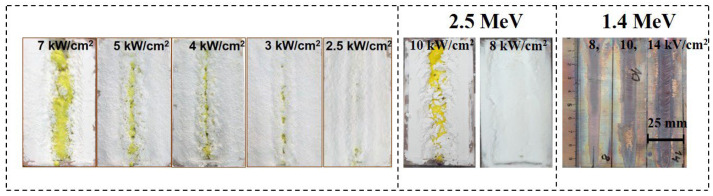
Photographs of ceramic samples synthesized under the exposed of electron fluxes with E = 1.4 MeV (P = 4—2.5 kW/cm^2^), E = 2.5 MeV (P= 10 and 8 kW/cm^2^), and traces of the impact of electron flows with E = 1.4 MeV (P = 8, 10, 14 kW/cm^2^) on the copper plate.

Photographs of ceramic samples in crucibles synthesized under the influence of electron fluxes with E = 1.4 MeV, E = 2.5 MeV at different power densities P are shown in Figure 17. The synthesis was carried out in the “without scanning” mode, which makes it possible to visually compare the results of the analysis of energy losses and synthesis.

Figure 17 shows photographs of YAG: Ce ceramics samples synthesized under the influence of electron flux with E= 1.4 MeV at various power levels. The samples were synthesized in a R2 mode, which visually presents the morphology of the formed samples. The electron beam was directed along the target, and the sample was formed in the shape of a strip against the background of the rest of the mixture.

At a power density of 7 kW/cm^2^ against the background of the mixture, a strip of YAG: Ce ceramics, characterized by its distinctive yellow color, was formed. Upon reducing the power density to 5–2.5 kW/cm^2^, the ceramics strip has become narrower.

From the provided images, it can be observed that during an exposure time of 10 s, ceramic samples in the form of characteristic yellow YAG:Ce rods are formed in the crucible by electron beams. At higher power (P), the rod-shaped samples are positioned on or near the irradiated surface. As the power (P) decreases, the formed samples can be concealed under a layer of mixture material. The depth at which the formed sample is situated within the mixture increases with higher energy (E). This trend corresponds to the conclusion made earlier regarding the dependence of the position of the region of maximum electron beam energy loss on E and P.

In the same figure, photographs of the traces of electron beam impact with E = 1.4 MeV in R2 mode on a thick copper plate are presented. In the experiment, the upper surface of the plate was placed at the same distance from the accelerator’s exit aperture as the external surface of the mixture during synthesis. The images clearly show that the width of the impact trace in the center of the image reaches 7–10 mm, with much higher power density at the center. At a power density (P) of 8 kW/cm^2^, only signs of oxidation are visible in the image, while at 12 kW/cm^2^, melting of the outer surface is observed. It is worth noting that the melting temperature of copper is 1085 °C, while the oxides being synthesized, such as A_2_O_3_ (2044 °C) and Y_2_O_3_ (2410 °C), have significantly higher melting temperatures. YAG:Ce ceramic synthesis, on the other hand, occurs under the same conditions of electron beam exposure at P < 4 kW/cm^2^, which can be explained by the differences in the dissipation of absorbed energy between metals and dielectrics.

Another noteworthy effect is observed. In the images shown in Figure 17, it can be seen that YAG:Ce ceramic synthesis occurs almost uniformly along the entire length of the crucible, which is moved relative to the electron beam. The trace of the electron beam impact on the copper plate has a variable width. As the plate is moved (or over time after the start of electron beam exposure), the trace expands and then remains constant over a longer length. This is explained by the fact that during radiation processing, the entire copper plate heats up to a certain threshold determined by the time it takes to establish equilibrium between the supplied and dissipated energy.

**Figure 18:**
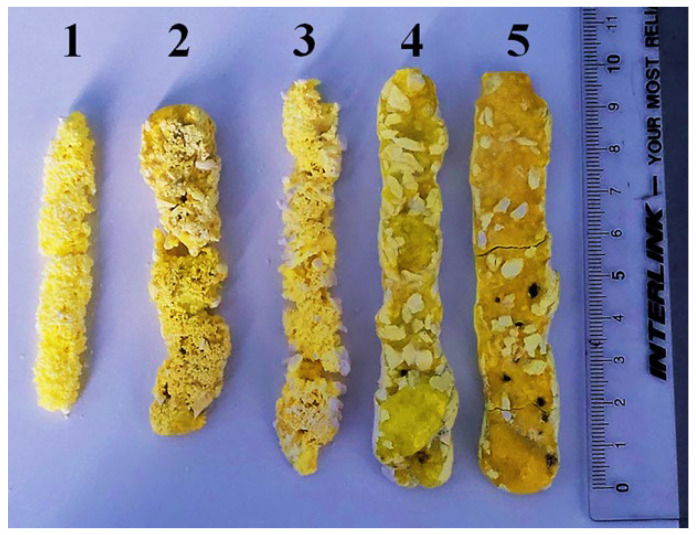
Photographs of YAG:Ce ceramic samples synthesized under the influence of electron beams with different values of E and P are as follows: 1—E = 1.4 MeV, P = 2.5 kW/cm^2^; 2—E = 2.0 MeV, P = 4 kW/cm^2^; 3—E = 2.5 MeV, P = 8 kW/cm^2^; 4—E = 2.0 MeV, P = 6 kW/cm^2^; 5—E = 2.5 MeV, P = 10 kW/cm^2^.

Figure 18 presents photographs of samples synthesized in R2 mode, removed from the crucibles. The first three were completely covered by mixture material, while the last two were open. All the samples are in the form of rods with different diameters. Sample 1 was synthesized with E = 1.4 MeV, P = 2.5 kW/cm^2^, 2 with E = 2.0 MeV, P = 4 kW/cm^2^, and 3 with E = 2.5 MeV, P = 8 kW/cm^2^. Sample 4 was only slightly covered by mixture material from the top (E = 2.0 MeV, P = 6 kW/cm^2^), and 5 was nearly completely exposed (E = 2.5 MeV, P = 10 kW/cm^2^).

Samples formed inside the mixture, at relatively low power density P, have a smaller length and a porous surface. Ceramic samples that have reached the surface of the mixture during ceramic formation have a solid continuous surface but are porous inside. It should be noted that the bright spots in the sample photographs are traces of the mixture, which are difficult to remove without damaging the sample.

As P decreases, the diameter of the forming sample decreases, and the solid rod turns into a dashed one. The smallest ceramic samples, in the form of rare, dashed particles with dimensions of approximately 3 mm in diameter and up to 10 mm in length, were obtained when exposed to electron beams with E = 1.4 MeV, P = 1.5 kW/cm². These samples are loose and disintegrate under slight pressure. Nevertheless, they exhibit the characteristic yellow color of YAG:Ce ceramics.

The experimentally obtained results of ceramic synthesis in mode R2 closely correspond to the patterns described above, obtained during the modeling of energy loss processes.

### 4.3. Redistribution of Absorbed Energy in the Developing Sample

As stated above, the energy deposition of high-energy electrons is unevenly distributed within the volume of the irradiated material. The maximum absorbed energy is located at a certain depth below the surface, depending on the electron energy. The positions of the energy loss maxima (dE/dx) for beams in the synthesis of YAG come at 2.8, 3.7, and 4.6 mm for energies of 1.4, 2.0, and 2.5 MeV, respectively. The energy loss in the maxima is 30–40% higher than at the surface and decreases to zero at the depth of the full range.

The energy transferred to the Irradiated material is converted into heat with non-uniform distribution within the material. In metals, the absorbed energy is converted into heat in less than 10^−12^ s, while in dielectric materials, including those we are studying, half of the energy is transferred within this time, and the other half within less than 10^−6^ s. After the electron beam’s impact ceases, thermal energy redistributes within the target. Heat from the hottest regions of the target shifts towards the colder layers, eventually equalizing the temperature throughout the target [52,53].

The characteristic length of the temperature front displacement (*l*) during a selected time (*t*) is determined by the following equation:

(1) $$l={\left(\frac{\lambda }{pC}\cdot t\right)}^{\frac{1}{2}},$$

where *λ* is thermal conductivity, *C* is specific heat capacity, and *ρ* is the material density (of the mixture). For the YAG ceramic mixture, *λ* = 0.15 − 0.16 W/mK [54], *C* ≈ 0.59 J/gK, and *ρ* = 1.15 g/cm^3^. During the 1-s synthesis, the displacement distance of the temperature front in the dielectric powder, such as YAG, is 0.28 mm. Therefore, within 1 s of radiation exposure, the temperature distribution remains relatively homogeneous when synthesizing a 1 cm long sample. Consequently, the resulting ceramic sample fully reflects the spatial structure of the absorbed energy distribution.

In contrast, heat energy redistributes differently in metals. The thermal conductivity of copper (401 W/mK) and steel (55 W/mK) is much higher than that of the materials used in the synthesis of the mixture, resulting in temperature front displacements of 50 mm and 20 mm, respectively, during the synthesis time. Thus, when electrons of the used energies (1.4–2.5 MeV) impact a copper or steel target, the temperature front can move a significant distance, sufficient to equalize the temperature within the volume. Since the electron penetration depth in copper and steel does not exceed 5 mm, all the heat reaches the surface, and any visible structural disturbances are concentrated there. This explains the difference in the formation of the radiation impact structure in ceramics and metals. In the ceramics of metal oxides and fluorides, the formation of a bulk structure is observed, whereas in metals at the energies of electrons employed, only surface disruption is observed (Figure 17).

Radiation synthesis, as shown in Section 2, is achieved in the studied materials by exposing the target to a flux of electrons with E = 1.4 MeV and power densities in the R1 mode ranging from 10 to 25 kW/cm^2^. Research has shown that ceramic synthesis occurs when the power density exceeds a characteristic threshold P_c_ for that material. Therefore, for each synthesized material, power densities were experimentally selected such that the synthesis reaction yield and morphology were sufficient for conducting experiments in the study and application of ceramics. It can be considered established that the synthesis of YAG ceramics is achieved using power densities above 2.5 kW/cm^2^ in the R2 mode (Figure 1 and Figure 17) or above 12 kW/cm^2^ in the R1 mode. Ranges have been identified within which aluminum magnesium spinel ceramics and alkaline earth metal fluorides ceramics are synthesized. To develop an understanding of the processes underlying radiation synthesis and to address technological issues, an assessment of the P_c_ power density values is necessary. Estimating P_c_ is complicated by two main factors. First, the distribution of absorbed electron energy is non-uniform within the volume, meaning that synthesis always occurs in the region of maximum energy loss, and synthesis thresholds are non-linearly related to the energy of the electrons used. Second, the synthesis threshold Pc may depend on the history of the materials used for synthesis. It is necessary to experimentally demonstrate the possible dependence of synthesis on the material’s history and understand the reasons behind it.

To date, the following approach seems reasonable for determining P_c_. During synthesis in the R2 mode, a rod-shaped specimen is formed in the mixture. As the power density P decreases, the samples cross-section decreases to a certain value. Below a certain P, the specimen becomes discontinuous, but its cross-sectional dimensions remain the same (Figure 18). Further reduction in P does not lead to ceramic formation. This value should be considered as the threshold.

## 5. Discussion

It has been experimentally demonstrated that by directly exposing a powerful flux of high-energy electrons with energies ranging from 1.4 to 2.5 MeV and power densities up to 40 kW/cm^2^, the synthesis of ceramics from powders of Mg fluorides, Ba, Al oxides, Y, Ga, Zn, Zr, Mg, and W is possible. This synthesis also extends to ceramics with new phase compositions, such as yttrium aluminum garnet, spinels, tungstates, and solid solutions of alkali earth metal fluorides. It has been established that ceramic synthesis can be achieved in all examined combinations of initial powders within the range of power densities from 10 to 25 kW/cm^2^ at E = 1.4 MeV (in “scanning” processing mode). It has been demonstrated that ceramic synthesis can be achieved under the specified conditions from powders with significantly different melting temperatures, ranging from 1263 °C (MgF_2_) to 2825 °C (MgO), including mixtures of powders. The synthesis of ceramics based on metal fluorides (T_mt_ 1300–1400 °C), metal oxides such as YAG (T_mt_ 2044–2410 °C), and MgAl_2_O_4_ spinel (T_mt_ 2044–2825 °C) is achievable when utilizing power densities exceeding 10 kW/cm^2^, even though the melting temperatures of the initial materials vary.

Radiation synthesis is successfully achieved from powder mixtures with particle sizes between 1–15 µm but is less effective when using nano- or sub-millimeter-sized particles. The combination of these observed regularities does not fit within the framework of thermal processes in radiation synthesis. It is hypothesized that ionization effects in the irradiated materials play a dominant role in radiation synthesis. When exposed to electron flux, the target material mix for synthesis is heated, and thermal processes contribute to the development of radiation-induced processes.

The high-speed synthesis of materials from refractory metal oxides in the presence of powerful fluxes of high-energy electrons suggests the existence of high-efficiency mutual element exchange between the mixture particles for the formation of new phases. Clearly, element exchange between crystalline particles is not possible within 1 s and is unlikely in the liquid phase after the instant melting of all particles. Element exchange between particles can occur within 1 s in an electron-ion plasma. It is known that relaxation times in the created ion–electron plasma have a magnitude of approximately 1–10 µs [55].

One can hypothesize that at high excitation densities in dielectric materials, the formation of radicals with high reactivity may occur, which will be capable of facilitating the creation of new phases corresponding to the specified stoichiometric composition.

### 5.1. Elementary Processes

The radiation fluxes used for synthesis provide a high density of electron–hole pairs. For example, in YAG at an electron energy of 1.4 MeV, a power density of 20 kW/cm^2^, an electron range of 0.2 cm, and a forbidden bandgap width of 8 eV in the metal oxides used for synthesis, approximately 4 × 10^22^ electron–hole pairs are created in 1 s in 1 cm^3^. This volume contains 4.7 × 10^21^ molecules in 6 × 10^20^ elementary cells. Thus, the concentration of electronic excitations capable of decaying into pairs of structural defects exceeds the number of molecules and cells by 1–2 orders of magnitude. The lifetime of electronic excitations before decay is within the range of τ_ep_ = 10^−9^ to 10^−10^ s. Consequently, at any moment of electron flux exposure, there are approximately 10^13^ electronic excitations within mutual distances of about 400 nm, comparable to the sizes of micro-particles, in the mixture. Therefore, there is a high probability of their localization on the surface, followed by decay under significantly different conditions through non-radiative recombination, leading to the formation of structural defects and radicals. Primary short-lived radiation defects transform into relatively long-lived pairs and radicals within the time range of 10^−9^ to 10^−3^ s. Therefore, the concentration of radicals can reach 10^19^ cm^−3^. At such a concentration, the medium under the influence of a powerful radiation flux can be considered an ion–electron plasma. Therefore, under the described conditions of radiation processing, the decay products of electronic excitations and radiolysis are capable of entering into mutual reactions, leading to the formation of new structural units.

It is known that electronic excitations in alkali halide crystals readily decay into pairs of structural defects with high efficiency. The yield of the excitation decay reaction increases with temperature and can reach 0.8. High efficiency in the decay into pairs of structural defects is also observed in crystals of alkaline earth metal fluorides. There are grounds to expect such high yields in other dielectric materials with a high degree of ionic bonding [55].

The processes of decay of electronic excitations into pairs of structural defects in metal oxide crystals have not been thoroughly studied. However, the phenomenon of coloration, i.e., the creation of color centers, is known. Even the formation of short-lived color centers in corundum tubes of gas discharge lamps is known. At high electron excitation densities, their decay into structural pairs implies the decomposition of the material into ions and radicals.

**Figure 19:**
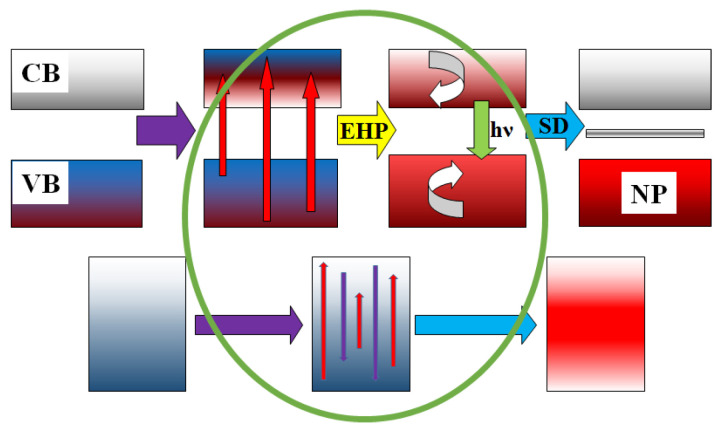
A schematic representation of the relaxation of excitation energy in dielectrics (at the top) and metals (at the bottom).

Thus, the entire set of processes for dissipating radiation energy in dielectric materials can be schematically represented (Figure 19) and described as follows: 99% of the energy of the electron flux with energies of 1.4–2.5 MeV is spent on material ionization, where electrons transition from the valence band (VB) to the conduction band (CB). The creation of an electron–hole pair (EHP) requires energy equal to 2–3 times the width of the bandgap; e.g., the creation time of EHP is no more than 10^−15^ s.

Then:

Relaxation to the lowest states (e_0_ to e_r_) occurs with the transfer of 0.5–0.7 energy to the lattice, and the relaxation time is less than τ = 10^−12^ s.

Decay of electronic excitations occurs, either radiative or non-radiative, into pairs of short-lived defects (SD), for example, Frenkel pairs and radicals (e_r_ F + H). The time range for these processes is from 10^−12^ s to 10^−9^ s, and some of the energy is transferred to the lattice. The decay of electronic excitations into SD pairs and their transformation into stable states is facilitated by the high temperature of the substance.

Recombination or transformation of primary pairs into stable complexes (F + H to F + V). The formation of new phases (NP) takes place in a time range from 10^−9^ to 10^−3^ s. Some of the energy is transferred to the emerging phase.

Cooling of the material (transfer of energy to the surrounding medium through radiation, thermal conductivity, convection) occurs over timescales longer than 1 s.

In metals, electronic excitations created under the influence of high-energy radiation disappear non-radiatively and without forming defects in timescales shorter than 10^−12^ s. The energy released during this process is immediately transferred to the lattice, heating the material.

Therefore, the main distinction between the energy dissipation processes of electronic excitations in dielectric and metallic materials lies in the existence of short-lived radiolysis products in dielectric materials. In metals, there are no processes associated with the decay of electronic excitations into pairs of defects or the formation of radicals—mobile intermediate components capable of participating in structural transformations during their existence.

**Figure 20:**
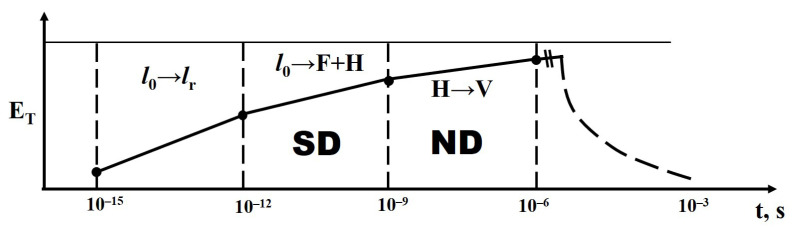
Schematic representation of the transfer of excitation energy to heating in a dielectric.

The transformation of structural phases after exposure to a radiation flux occurs with the heating of the irradiated material. Figure 20 schematically represents the dependence of the amount of energy transferred to the material’s heating during the relaxation of electronic excitations and structural phase transformations. At least half of all absorbed radiation energy is transferred to the lattice during the relaxation of created electronic excitations to their lowest states in a time period of τ = 10^−12^ s. The creation of primary short-lived defects occurs with the transfer of a portion of the energy from relaxed electronic excitations, excitons, to the lattice within a time period of τ = 10^−9^ s. This portion can constitute approximately half of the exciton’s energy. Primary defects transform into stable radicals, which form a new phase within timescales of τ = 10^−3^ s. Transformations of stable radicals are also possible within timescales of τ = 10^3^ s.

When exposed to a stationary electron flux, the target must heat up over time to temperatures at which thermal equilibrium is reached between the processes of heat input and heat exchange with the surrounding environment. Under the conditions employed in radiation synthesis, heat exchange occurs between the heated target, the charge, and the surrounding environment, including thermal radiation, convective exchange with the air, and heat transfer into the copper crucible. As evident from the graph (Figure 17), when the copper plate is displaced relative to the electron beam (Mode R2) at a velocity of 1 cm/s, the width of the trace increases, but it remains unchanged after 1–2 s. Consequently, thermal equilibrium between energy input and heat dissipation is reached within 1 s of electron beam exposure. A similar effect occurs when irradiating the charge of synthesized ceramics. The heating of the charge of synthesized ceramics precedes the displacement of the beam. Therefore, synthesis takes place in an environment that has already reached a maximum temperature. Since the efficiency of the decay of electronic excitations into structural pairs increases with temperature, the efficiency of radiolysis also increases and becomes constant, characteristic of the specific material.

### 5.2. On the Threshold of Synthesis

To implement radiation synthesis, it is necessary to create such a density of radicals in the particles of the substances used that can facilitate the exchange of elements between the particles. The lifetime of the radicals probably spans a wide range from 10^−9^ to 10^−3^ s. Therefore, the choice of the power density of the electron beam that provides a sufficient density of radicals for synthesis can only be determined experimentally at this time. Clearly, for each material or combination of materials, there exist characteristic thresholds for the power density of the electron beam above which synthesis can be realized. However, estimating these synthesis threshold values is only possible under specific conditions of radiation processing: electron energy, spatial distribution of the beam flux, irradiation modes (with scanning or without scanning).

In the conditions we are using for high-energy electron beam impact on the synthesis layer, there is a pronounced non-uniform distribution of absorbed energy, leading to the creation of a non-uniform density of electronic excitations. The probability of radiation synthesis is higher in the region of maximum electronic excitation density. In this region, synthesis will primarily occur as the power density of the electron beam increases. As the power density of the beam increases, this region expands (Figure 17).

Therefore, according to the presented understanding of ceramic synthesis processes under the influence of high-energy electron beams, there should be a threshold power density P_c_ for each material above which the process of forming a new phase or morphology becomes possible. Knowledge of this threshold is crucial for understanding the dependence of synthesis efficiency on the energy and morphological properties of the initial materials. At present, it seems reasonable to adopt the following approach to determine P_c_: during synthesis in the R2 mode, a rod-shaped sample is formed in the layer. As the power density P decreases, the cross-sectional area of the sample decreases until it reaches a certain value. Below a certain P, the sample becomes discontinuous, but the dimensions of its cross-section remain the same (Figure 18). In the synthesis of YAG samples, the minimum diameter of the sample reaches 4–5 mm, with a minimum length of 10 mm. With further reduction in P, ceramic formation is not observed. This value should be considered as the threshold.

## 6. Conclusions

The synthesis of ceramics through the direct impact of a powerful flux of high-energy electrons on mixtures of dielectric powders with high melting temperatures has been achieved. These powders include MgF_2_ (1263 °C), BaF_2_ (1368 °C), WO_3_ (1473 °C), Ga_2_O_3_ (1725 °C), ZnO (1975 °C), Al_2_O_3_ (2044 °C), Y_2_O_3_ (2410 °C), ZrO_2_ (2715 °C), MgO (2825 °C), Y_3_Al_5_O_12_, Y_3_Al_x_Ga_5−x_O_12_, MgAl_2_O_4_, ZnAl_2_O_4_, MgWO_4_, ZnWO_4_, Ba_x_Mg(_2−x_) F_4_, Ba_x_Mg(_2−x_) F_4_: W, Y_3_Al_5_O_12_: Gd, Ce, Cr, Eu, Er, and MgAl_2_O_4_: Ce, Cr, Eu, Er. The formation of ceramics from these compositions suggests the method’s universality. It can be asserted at this time that radiation synthesis has produced ceramics based on YAG (Y_3_Al_5_O_12_), spinel (MgWO_4_), and solid solution (BaMgF_4_) with the characteristic properties of these materials. Expanding the range of synthesized materials and establishing criteria for proving the implementation of radiation ceramic synthesis are necessary.

The main properties of radiation synthesis are as follows. Synthesis is achieved:

- Solely through radiation energy;
- Exclusively from mixture materials;
- Without the addition of any other materials to facilitate synthesis;
- In less than 1 s.

The high synthesis speed allows for the rapid execution of necessary improvements, conducting series of experimental studies to optimize synthesis, which is particularly important for multi-component luminescent materials.

The set of processes enabling the radiation synthesis of dielectric and metallic materials differs. Processes in dielectric and metallic materials vary due to relaxation of excited states after ionization by the radiation flux. It is essential to develop an understanding of radiation-induced processes, radiolysis, structural (phase) transformations, and the formation of new materials with new properties stimulated by high radiation flows.

Radiation exposure efficiently facilitates the mutual transfer of elements between charge particles of mixture materials and the formation of a new phase with the same morphology, as well as the introduction of additional elements from added particles (activation). Powerful radiation exposure ensures high-efficiency mixing of particle elements from the initial materials used.

Ceramic synthesis occurs when a certain threshold power density of the beam P_c_ is exceeded. Knowledge of this threshold is crucial for understanding the dependence of synthesis efficiency on the energetic and morphological properties of the initial substances. Experimental determination of this threshold and its relationship with the history of the initial substances is necessary.

Synthesis efficiency depends on the history of the initial materials, especially their dispersity. The best results are obtained from powders with particle sizes ranging from 3 to 10 µm.

Samples synthesized in a powerful electron beam have a solid shell and a porous internal structure. The structural and luminescent properties of the external and internal layers of ceramics are not different. The presence of pores affects the transparency of samples, causing light scattering. To obtain transparent ceramics, a method to reduce porosity needs to be found.

## Figures and Tables

**Table 1:** The results of studying the phase composition of magnesium fluoride samples synthesized at a power density of 20 kW/cm^2^.

Main Phase	Secondary Phase
MgF_2_	It is possible that WO_3_ (tungsten trioxide) is present, with an impurity phase of 2–3%
BaF_2_	Impurity phase of 2–3%
BaMgF_4_	Impurity phase of 2–3%

**Table 2:** Efficiency of radiation ceramic synthesis.

Continuity No	Sample	Description	Power P, kW	Mixture Weight, g	Mass Loss, %	Yield Sample/Mixture, %
407	Al_2_O_3_	F-800	26	40.53	1.8	94.9
493	Al_2_O_3_	nano	25	43.33	17.2	4.5
519	BaF_2_	(K14)	15	88.12	0.8	76
516	MgF_2_	(K13)	15	42.52	0.4	99.3
485	MgO	(K11)	25	26.94	5.7	90.9
486	MgO	G	35	39.94	90	4.6
388	MgO	MgO (1)	8	22.22	1.76	20.3
397	MgO	MgO (2)	8	14.19	46.65	0.0
528	WO_3_	K12	17	95.4	4.95	77.2
439	WO_3_	WO_3_ (1)	24	100.38	35.02	46.1
490	Y_2_O_3_		25	56.01	1.3	91.9
495	ZrO_2_	ZrO_2_ (2)	5	56.32	52.1	0
146	ZrO_2_	ZrO_2_ (1)				80
474	Ga_2_O_3_		17	53.34	0.8	95.7
525	MgAl_2_O_4_: Eu	Al_2_O_3_ (K7), Mg*O* (K11)	25	36.58	0.4	99
384	MgAl_2_O_4_: Eu	MgO (1), Al_2_O_3_ (F-800)	25	41.31	51.9	23.1
377	MgAl_2_O_4_: Eu	MgO K11, Al_2_O_3_ (F-800)	25	33.95	1.1	96.9
383	MgAl_2_O_4_: Eu	MgO (1), Al_2_O_3_ (F-800)	25	39.23	53.5	34.7
525	MgAl_2_O_4_: Eu	MgO (1), Al_2_O_3_ (F-800)	25	41.31	51.9	23.1
521	BaMgF_4_	BaF_2_ (K14) MgF_2_(K13)	15	81.57	0.5	92.8
335	Y_3_Al_5_O_12_: Ce	Al_2_O_3_ (alund) Y_2_O_3_ (ITO-B)	25	35.2	0.7	97.8
338	Y_3_Al_5_O_12_: Ce	Al_2_O_3_ (nano) Y_2_O_3_ (ITO-B)	25	33.38	32.2	53.02
505	Y_3_Al_5_O_12_: Ce	Al_2_O_3_ (K7), Y_2_O_3_ (K6)		25	45.9	99.1
504	ZnAl GaO_4_	ZnO K9, Al_2_O_3_ (K7) Ga_2_O_3_ (K9)	22	42.08	0.9	99.3
526	ZnAl_2_O_4_	ZnO K9, Al_2_O_3_ (K7)	25	43.3	1.1	98.3
512	MgWO_4_	MgO (K11)	18	73.53	0.7	97.4
444	MgWO	MgO (1)	15	45.72	30.3	42.20

## Data Availability

The data presented in this study are available on request from the corresponding author.
